# Astrocytes and Adenosine A_2*A*_ Receptors: Active Players in Alzheimer’s Disease

**DOI:** 10.3389/fnins.2021.666710

**Published:** 2021-05-13

**Authors:** Cátia R. Lopes, Rodrigo A. Cunha, Paula Agostinho

**Affiliations:** ^1^Center for Neuroscience and Cell Biology, Coimbra, Portugal; ^2^Faculty of Medicine, University of Coimbra, Coimbra, Portugal

**Keywords:** astrocyte reactivity, amyloid-β protein, synaptic plasticity, cognitive deficits, adenosine A_2*A*_ receptors, Alzheimer’s disease

## Abstract

Astrocytes, through their numerous processes, establish a bidirectional communication with neurons that is crucial to regulate synaptic plasticity, the purported neurophysiological basis of memory. This evidence contributed to change the classic “neurocentric” view of Alzheimer’s disease (AD), being astrocytes increasingly considered a key player in this neurodegenerative disease. AD, the most common form of dementia in the elderly, is characterized by a deterioration of memory and of other cognitive functions. Although, early cognitive deficits have been associated with synaptic loss and dysfunction caused by amyloid-β peptides (Aβ), accumulating evidences support a role of astrocytes in AD. Astrocyte atrophy and reactivity occurring at early and later stages of AD, respectively, involve morphological alterations that translate into functional changes. However, the main signals responsible for astrocytic alterations in AD and their impact on synaptic function remain to be defined. One possible candidate is adenosine, which can be formed upon extracellular catabolism of ATP released by astrocytes. Adenosine can act as a homeostatic modulator and also as a neuromodulator at the synaptic level, through the activation of adenosine receptors, mainly of A_1_R and A_2*A*_R subtypes. These receptors are also present in astrocytes, being particularly relevant in pathological conditions, to control the morphofunctional responses of astrocytes. Here, we will focus on the role of A_2*A*_R, since they are particularly associated with neurodegeneration and also with memory processes. Furthermore, A_2*A*_R levels are increased in the AD brain, namely in astrocytes where they can control key astrocytic functions. Thus, unveiling the role of A_2*A*_R in astrocytes function might shed light on novel therapeutic strategies for AD.

## Adenosine Signaling and Astrocyte-Neuron Communication

One pathway underlying astrocyte-neuron interactions in CNS is the purinergic signaling, mainly operated by ATP and adenosine that constitute two superimposed signaling systems (Agostinho et al., 2020). ATP released by astrocytes, a subtype of glial cells, is a significant source of adenosine in the brain, being the extracellular adenosine levels regulated by a set of ectonucleotidases, in particular by astroglial ecto-5′-nucleotidase (CD73), which is the last and rate-limiting step in the extracellular formation of ATP-derived adenosine (Brisevac et al., 2015). Moreover, adenosine can also be released directly *via* equilibrative nucleoside transporters (ENT), such as ENT-1 and ENT-2, being the intracellular levels of this nucleoside controlled by the activity of adenosine kinase (ADK), which is mainly expressed in astrocytes and metabolize the conversion of adenosine into AMP (Boison et al., 2010). Adenosine is considered both a neuromodulator and a stress signal, and its functions are mediated by four subtypes of adenosine receptors: A_1_R, A_2*A*_R, A_2*B*_R, and A_3_R, which are G-protein-coupled receptors, each with a unique pharmacological profile. These metabotropic receptors can recruit different enzymatic activities and/or changes of ion channels function to mediate modulatory actions, with A_1_R and A_3_R being considered inhibitory and A_2*A*_R and A_2*B*_R as facilitatory (reviewed in Cunha, 2016, 2019; Agostinho et al., 2020). Briefly, the activation of A_1_R and A_3_R, through the action of Gi proteins, inhibits adenylyl cyclase (AC) activity and leads to increased activity of phospholipase C that further triggers its downstream signaling pathways, whereas A_2*A*_R and A_2*B*_R activation, through Gs proteins instigation, increases the production of cAMP to activate protein kinase A (PKA) signaling pathways (Jacobson and Gao, 2006; Cunha, 2016; Agostinho et al., 2020).

Adenosine’s effects in the brain are mostly mediated by A_1_R and A_2*A*_R, which prime role is the modulation of synaptic activity, interfering with information transmission within neuronal circuits (reviewed in Fredholm et al., 2005; Cunha et al., 2008; Agostinho et al., 2020). Both A_1_R and A_2*A*_R are mostly located in synapses in particular in excitatory (glutamatergic) synapses, although they are also present in gamma-aminobutyric acid (GABA)ergic, cholinergic, dopaminergic, serotoninergic, or noradrenergic synapses (reviewed in Cunha, 2016). In excitatory synapses, adenosine under basal conditions can inhibit synaptic transmission *via* A_1_R-mediated activity mainly located in presynaptic terminals (Rebola et al., 2003, 2005). In contrast, A_2*A*_R are only recruited upon higher frequencies of nerve stimulation, triggering plastic changes of synaptic efficiency that enhance glutamate release as well as *N*-methyl-D-aspartate receptor (NMDAR) activation (Rebola et al., 2008), in order to facilitate synaptic plasticity (reviewed in Cunha, 2016). Synaptic plasticity, including long-term potentiation (LTP) and long-term depression (LTD), is considered the neurophysiological basis of memory (Martin et al., 2000; Neves et al., 2008). Accordingly, a study using optogenetic tools showed that A_2*A*_R activation, through a phospho-CREB signaling in the hippocampus is sufficient to impair memory function (Li et al., 2015). Moreover, there is evidence supporting a correlation between the adenosine sources and the type of adenosine receptors that are activated, insofar as it was described that A_1_R are mainly activated by the tonus of adenosine, formed from the catabolism of ATP released from astrocytes (Pascual et al., 2005) and by postsynaptic adenosine efflux (Lovatt et al., 2012), whereas adenosine derived from synaptically-released ATP, due to CD73 action, mainly activates neuronal, mainly postsynaptic, A_2*A*_R (Cunha et al., 1995; Rebola et al., 2008; Augusto et al., 2013; Carmo et al., 2019; Gonçalves et al., 2019).

Adenosine can also act as an astrocytic modulator, regulating astrocytic metabolism (Lemos et al., 2015), Ca^2+^ waves (Kawamura and Kawamura, 2011; Kanno and Nishizaki, 2012), and neurotransmitter uptake ability (Nishizaki et al., 2002; Cristóvão-Ferreira et al., 2013; Matos et al., 2013). In relation to adenosine signaling in astrocytes, it has been documented that all the different subtypes of adenosine receptors are present in astrocytes, although A_1_R and A_2*A*_R have been the most studied (Daré et al., 2007; Boison et al., 2010; Agostinho et al., 2020). Under physiological conditions, the role of adenosine signaling in astrocytes is mostly hypothetical, since it is based on the effects of A_1_R and A_2*A*_R in cultured astrocytes; additionally, the role of these receptors in pathological conditions, controlling the morpho-functional changes of reactive astrocytes, seems to be better supported by experimentation, although still far from being established. Among the functions known until now, A_1_R in astrocytes mediate an immunosuppressive effect, whereas astrocytic A_2*A*_R can trigger transcriptional deregulation (Paiva et al., 2019) and mediate astrocyte reactivity (Brambilla et al., 2003; Ke et al., 2009), control glutamate release and consequently synaptic transmission (Nishizaki et al., 2002; Cervetto et al., 2017), regulate glutamate uptake by controlling the levels of glutamate transporters and the activity of Na^+^/K^+^-ATPase (Nishizaki et al., 2002; Matos et al., 2013). Moreover, it was shown that astrocytic A_2*A*_R and Gs-coupled signaling regulate memory consolidation in mice (Orr et al., 2015). Altogether, these findings highlight the relevant role of astrocytic A_2*A*_R in regulating synaptic plasticity and memory, suggesting that A_2*A*_R in astrocytes might also be a good candidate to normalize memory in case of pathology associated with cognitive deficits, such as in Alzheimer’s disease (AD).

## Astrocytes’ Role in Alzheimer’s Disease

In the last years, the neurocentric view of AD, which considers neurons as the major players in this neurodegenerative process, has been challenged due to increasing evidences supporting a role of astrocytes in this age-related disease. Although the pathological potential of astrocytes in AD was first recognized in 1910 by Alois Alzheimer, who described the presence of glial cells in close association with dying neurons and reported that glial cells made part of senile plaques (reviewed in Serrano-Pozo et al., 2011; Rodríguez-Arellano et al., 2016), the role of astrocytes in AD pathology has been underexplored.

Alzheimer’s disease pathology is associated with an abnormal production of amyloid-beta (Aβ) peptides that accumulate extracellularly over time, as amyloid plaques. Aβ peptides are generated through the sequential proteolytic processing of amyloid-beta precursor protein (AβPP) by β-secretase (BACE1) and γ-secretase. Other neuropathological features of AD are the intracellular formation of intracellular neurofibrillary tangles (NFT) composed of hyperphosphorylated tau protein, together with synaptic dysfunction and loss that progress to neuronal death (Haass and Selkoe, 2007; Selkoe and Hardy, 2016). Several studies have shown that in early AD, corresponding to the first symptoms of cognitive deficits, soluble Aβ oligomers trigger an extensive proliferation of astrocytes with a reactive phenotype. Reactive astrocytes, besides functional changes, exhibit alterations of their morphology that encompass a hypertrophy of the cell soma and the shortening and/or thickening of astrocytic processes, as well as the upregulation of several astrocytic structural proteins, such as glial fibrillary acidic protein (GFAP), or cell signaling proteins, like S100β (Ceyzériat et al., 2018; Escartin et al., 2021). Astrocyte reactivity usually persists and aggravates over time and parallels Aβ deposition, being common to observe reactive astrocytes surrounding amyloid plaques in the hippocampus of AD mouse models (Olabarria et al., 2010), as well as in human AD brains (Kashon et al., 2004). Astrogliosis *in vivo* can be assessed by positron-emission tomography detection of ^11^C-deuterium-L-deprenyl (^11^C-DED); deprenyl is a selective inhibitor of monoaminoxidase-B (MAO-B) localized in astrocytes, and an increase in the ^11^C-DED signal reflects astrocyte hypertrophy (Fowler et al., 1997; Verkhratsky et al., 2019). On the other hand, the astrocytes located away from amyloid plaques are usually atrophic (Rodríguez et al., 2009; Olabarria et al., 2010). Astrocyte atrophy is detected in several AD mice models as well in in postmortem tissues of patients with advanced (Braak V–VI) stages of AD (see review, Verkhratsky et al., 2019), corresponding to a reduction of astrocytes territories with a decrease in coverage of synaptic contacts and other neuronal structures, and is accompanied by a loss of astrocyte function. These morphofunctional astrocyte changes can also lead to early cognitive deficits through dwindling neuronal support and synaptic dysfunction (Verkhratsky et al., 2019).

Studies performed in mouse models of AD have provided important information about the astrocytic alterations along AD progression. A reduction of volume and surface area of astrocytes, and a decrease in their processes, corresponding to a process of astrodegeneration, has been observed in AD mouse models. Triple transgenic AD mice, 3×TgAD, exhibit astrodegeneration before Aβ deposition in the medial prefrontal cortex, entorhinal cortex, and hippocampus (Yeh et al., 2011; Kulijewicz-Nawrot et al., 2012; Rodríguez et al., 2014). Astrocyte atrophy was also reported in the hippocampus of other transgenic AD (PDAPP) mouse model at early phases, before formation of amyloid plaques (Beauquis et al., 2014). In contrast, at later stages of AD, the presence of Aβ deposits triggers a secondary astroglial response, crresponding to a robust reactive astrogliosis in areas surrounding the amyloid plaques (Olabarria et al., 2010; Rodríguez-Arellano et al., 2016; Verkhratsky et al., 2016). This prompts the hypothesis that both this distal atrophy of astrocytes and the proximal astroglial reactivity contribute to the development of AD pathology.

Although, astrogliosis was long considered a broad secondary reaction to pathological conditions, reactive astrocytes can cause harmful effects to other brain cells, either as a consequence of loss of their normal homeostatic functions or due to a gain of toxic functions, linked to a decreased capacity of these cells to remove pathogens and amyloid proteins, in particular Aβ, or to phagocyte dystrophic synapses and cell debris (Barreto et al., 2011; Rodríguez-Arellano et al., 2016; Garwood et al., 2017). There is evidence that reactive astrocytes contribute to amyloid plaque formation, and consequently AD pathology, by different mechanisms, including: (i) hampered phagocytic capacity that is mediated by actin-regulated phagocytosis and/or by several membrane receptors (e.g., lipoprotein receptor-related ligand 1, receptor for advanced glycation end products (RAGE), scavenger receptors); (ii) reduced capacity to degrade the internalized Aβ, which involves the production of proteases, such as neprilysin, insulin-degrading enzyme, and enzymes of the ubiquitin-proteasome system (reviewed in Wyss-Coray et al., 2003; Miners et al., 2008; Ries and Sastre, 2016; Rodríguez-Arellano et al., 2016); and (iii) increased Aβ production by astrocytes due to BACE1 upregulation and, subsequent, amyloidogenic processing of AβPP triggered by proinflammatory conditions, mainly by IL-1β and TNF-α (Blasko et al., 2000; Sastre et al., 2003; Zhao et al., 2011). Although it was reported that reactive astrocytes produced Aβ in less quantity than neurons, astrocytes mostly produce N-truncated Aβ species, which are highly prone to aggregation and more toxic than the species produced by neurons (Oberstein et al., 2015; Frost and Li, 2017). Besides having a key role in amyloid pathology, reactive astrocytes can also participate in tau pathology (reviewed in Kovacs, 2020). A recent study reported that hippocampal astrocytes in the dentate gyrus of AD patients exhibit hyperphosphorylated tau, and this abnormal tau impairs Ca^2+^ oscillation and mitochondria motility, distribution, and function in astrocytes, contributing also to reduce: (i) adult neurogenesis; (ii) parvalbumin-expressing neurons; (iii) inhibitory synapses; and (iv) hilar gamma oscillations, which were accompanied by a weakened spatial memory performance (Richetin et al., 2020). Remarkably, since the presence of tau is not detectable in astrocytes under physiological conditions, the origin of this protein in astrocytes in AD-like conditions has been speculated, being proposed that during AD progression, tau translation might occur from mRNA present in astrocytes and astrocytic uptake of extracellular tau, released in the interstitial fluid by neurons, as well as intercellular propagation of tau through exosomes (see Richetin et al., 2020, and references within). In agreement, it was reported that astrocyte reactivity correlates with NFT density in the brains of AD patients (Overmyer et al., 2000; Gallardo and Holtzman, 2019). These findings contribute to sustain that astrogliosis is more directly associated with NFT, and hyperphosphorylated tau, since reactive astrocyte responses increase linearly with NFT burden and distribution, but not with amyloid pathology that tends to reach a plateau (Ingelsson et al., 2004; Serrano-Pozo et al., 2011).

Since the 1990s, several studies pointed out that astrocytes enwrap synaptic terminals and exchange information with them, responding to synaptic activity and regulating synaptic transmission (Parpura et al., 1994; Araque et al., 1999; Halassa et al., 2007; Covelo and Araque, 2016). Thus, astrocyte reactivity, due to Aβ accumulation and tau dyshomeostasis, may compromise synaptic plasticity and consequently memory. Accordingly, immunohistochemistry studies, assessing GFAP or S100β upregulation and distribution in AD patient’s brain, reported that the degree of astrogliosis is correlated with cognitive decline (Beach and McGeer, 1988; Mrak et al., 1996; Kashon et al., 2004). Studies performed in cultured astrocytes exposed to Aβ peptides showed that these cells became reactive and had a decreased glutamate uptake capacity, due to a downregulation of glutamate transporters, mainly of GLT-1 (Matos et al., 2012b; Zumkehr et al., 2015), being also shown that Aβ_1–42_ peptide promotes GLT-1 internalization (Scimemi et al., 2013). These alterations could contribute not only to excitotoxicity but also to impair synaptic plasticity occurring in AD conditions. Accordingly, it was reported that genetic reduction of GLT-1 levels accelerates the onset of cognitive deficit in a double (AβPPswe/PS1ΔE9) transgenic AD mouse model (Mookherjee et al., 2011), whereas the pharmacological upregulation of GLT-1 ameliorates the pathological tau accumulation, restores synaptic proteins and rescues cognitive decline, with minimal effects on Aβ pathology, in 3xTgAD mice (Zumkehr et al., 2015).

Moreover, Aβ accumulation causes an increased astrocytic excitability, mediated by sporadic Ca^2+^ signals that spread over to other astrocytes in the form of Ca^2+^ waves, which were shown to impact on synaptic transmission (Kuchibhotla et al., 2009). The hyperactive Ca^2+^ signaling might trigger an aberrant release of gliotransmitters, mainly through exocytosis involving the fusion of SNARE proteins of vesicles with the plasma membrane (Araque et al., 1999; Genoud et al., 2006). These alterations in astrocyte excitability and in gliotransmitters release (e.g., ATP, glutamate, D-serine) sustain the possibility of an abnormal metaplasticity, i.e., the regulation of synaptic plasticity by astrocytes, which might underlie the cognitive deficits observed in most mouse models of AD (Jones, 2015). The increased levels of glutamate and of Aβ oligomers, occurring in early AD phases, affect NMDAR subunits, NR2A and NR2B, which are crucial for synaptic plasticity, being the increased NR2B levels particularly associated with LTP inhibition and metaplasticity inversion in hippocampal slices of a transgenic AD mouse model (AβPP23) with impaired spatial working memory (Balducci et al., 2010). Moreover, soluble Aβ oligomers can engage the astrocytic α7 nicotinic acetylcholine receptors to induce glutamate release from astrocytes that in turn activate extrasynaptic NMDAR in neurons, causing a reduction in miniature excitatory postsynaptic currents (Talantova et al., 2013). In line with these findings, it is believed that the beneficial role of memantine (an NMDAR antagonist), in patients with moderate to severe AD, is mainly mediated through the blockade of extrasynaptic NMDAR activated by excess glutamate (Reisberg et al., 2003). GABA is the major inhibitory transmitter in the adult mammalian brain, and AD patients have increased GABA levels in their cerebrospinal fluid (Samakashvili et al., 2011) and antagonists of GABA_*A*_ receptors improve hippocampal LTP and memory in an AD (APP/PS1) mouse model (Yoshiike et al., 2008). In line with these findings, it was reported that hippocampal reactive astrocytes of different AD mouse models (APP/PS1 and 5xFAD) excessively produce GABA and release GABA abundantly through bestrophin 1 (Best1), a channel that in non-reactive astrocyte mediates the release of glutamate (Jo et al., 2014). Moreover, these authors also showed that the abnormal release of GABA by reactive astrocytes reduces the spike probability of granule cells by acting on presynaptic GABA receptors, leading to an impairment of synaptic plasticity and memory in AD mouse models (Jo et al., 2014). Another study also reported that in human AD brains, hippocampal astrocytes have a higher GABA content, and that 5xFAD mice modeling AD have astrocytes also with higher GABA levels and release through astrocyte-specific GABA transporters, GAT3/4 (Wu et al., 2014).

In conclusion, astrocytes support neuronal function, in particular synaptic plasticity, in many ways, and it is plausible that the dysfunction of these glial cells contributes to cognitive deficits associated with AD. In the last years, most of the therapies developed for AD were directed to avoid Aβ formation and accumulation or to normalize synaptic plasticity, as, for example, by inhibiting acetylcholinesterase to normalize acetylcholine levels in the synaptic cleft and by NMDAR antagonism. These strategies have been shown to be little effective, thus there is a need to find novel targets to delay the onset of synaptic and memory deficits in AD (Mangialasche et al., 2010; Morsy and Trippier, 2019).

A possible valid target for AD management is astrocytes, more precisely the manipulation of their functions, and a promising candidate to interfere with the ability of astrocytes to control synaptic function and memory is adenosine receptors, in particular adenosine A_2*A*_R. This stems from observations that: (i) A_2*A*_R are located in astrocytes, where they critically control Na^+^/K^+^-ATPase (Matos et al., 2012b, 2013), the main energizing system to sustain membrane-dependent processes in astrocytes; (ii) astrocytic A_2*A*_R control glutamate uptake by GLT-1, a process de-regulated in an AD mouse model (Matos et al., 2013); and (iii) astrocytic A_2*A*_R are upregulated in AD animal models and patients (Matos et al., 2015, 2012b; Orr et al., 2015). This is of particular importance in view of the convergence of epidemiological and animal studies showing that caffeine intake is inversely correlated with memory deterioration in aging and in AD, an effect mimicked by the selective A_2*A*_R blockade (Cunha and Agostinho, 2010; Agostinho et al., 2020). Noteworthy, our group showed that the pharmacological activation of A_2*A*_R (Pagnussat et al., 2015) or the optogenetic activation of neuronal A_2*A*_R intracellular signaling in the hippocampus is actually sufficient to impair memory (Li et al., 2015), and these receptors in astrocytes were also shown to regulate memory processes, since the genetic ablation of astrocytic A_2*A*_R enhances memory performance of aged mice modeling AD (Orr et al., 2015).

## Adenosine A_2*A*_R Signaling in AD: What Is the Role of Astrocytic A_2*A*_R?

A_2*A*_R signaling, besides having a discrete role in normal brain function, is mainly able to modulate the development or progression of several brain diseases, including AD (Gomes et al., 2011; Cunha, 2016; Franco and Navarro, 2018). Accordingly, the antagonism of A_2*A*_R has been shown to confer neuroprotection in several injurious and pathological brain conditions and can recover memory deficits in animal models of AD, which prompts A_2*A*_R as a therapeutic target for this disease (Arendash et al., 2006; Dall’lgna et al., 2007; Canas et al., 2009; Laurent et al., 2016; Viana da Silva et al., 2016; Silva et al., 2018). The neuroprotective effect of A_2*A*_R blockade against different brain pathologies, is mimicked by caffeine (Arendash et al., 2006; Dall’lgna et al., 2007; Takahashi et al., 2008; Espinosa et al., 2013; Laurent et al., 2014; Cunha, 2016). The regular consumption of caffeine/coffee, a non-selective adenosine receptor antagonist that at dose usually consumed by humans (around 200–300 mg caffeine or 3–5 cups of coffee/day) acts particularly as an A_2*A*_R antagonist (Fredholm et al., 2005), is inversely correlated with the incidence of AD and later dementia in humans (Maia and de Mendonça, 2002; Eskelinen et al., 2009), and is also protective against cognitive decline in AD mouse models (Arendash et al., 2006; Dall’lgna et al., 2007; Cao et al., 2009; Han et al., 2013; Chen, 2014; Laurent et al., 2014). Curiously, it was also reported that caffeine enhances the consolidation of long-term memories in humans, 24 h after a one-dose (200 mg) administration (Borota et al., 2014). Moreover, our group demonstrated that in rodent AD models, consisting in the intracerebroventricular Aβ injection, both caffeine and the selective A_2*A*_R antagonist SCH58261 prevent Aβ-induced cognitive impairments and synaptotoxicity (Dall’lgna et al., 2007; Cunha et al., 2008; Canas et al., 2009). A similar protective effect of the A_2*A*_R antagonist SCH58261 was also observed in AD transgenic (3xTgAD and APP/PS1) mouse models (Viana da Silva et al., 2016; Silva et al., 2018). These data were complemented with studies in rat primary-cultured neurons where it was observed that the pharmacological A_2*A*_R blockade attenuates Aβ-induced neuronal death through a reduction in A_2*A*_R-mediated p38 mitogen-activated protein kinase (MAPK) activation and preservation of hippocampal synaptosome function (Canas et al., 2009).

All these findings reporting that the blockade of A_2*A*_R prevents synaptic dysfunction and cognitive deficits, mainly memory loss, in conditions of AD, support that the modulation of synaptic function may constitute an interesting strategy to improve memory dysfunction related to neurodegenerative processes (Coleman et al., 2004; Wishart et al., 2006; Canas et al., 2018).

In accordance with the putative role of A_2*A*_R as a therapeutic target in AD, it was reported that A_2*A*_R levels are increased in the hippocampus of AD patients, in astroglial cells (Angulo et al., 2003; Orr et al., 2015), and also in the frontal cortex of AD (Albasanz et al., 2008). A recent study showed that aging also caused a significant upsurge of A_2*A*_R in hippocampal neurons of aged humans, a phenotype aggravated in AD patients (Temido-Ferreira et al., 2020). Regarding A_1_R, there are evidences from positron emission tomography (PET) studies that their levels are decreased in the hippocampus of AD patients (Fukumitsu et al., 2008), whereas increased levels of A_1_R were detected in postmortem AD frontal cortex (Albasanz et al., 2008) and in degenerating neurons with neurofibrillary tangles and in dystrophic neurites of senile plaques (Angulo et al., 2003). Likewise, in AD mice and in aged mice, a cortical and hippocampal upsurge of A_2*A*_R was reported mainly in glutamatergic synapses (Diógenes et al., 2007; Canas et al., 2009; Costenla et al., 2011), and recently, A_2*A*_R overexpression was reported to be sufficient to drive age-like memory impairments in young rats and to uncover a hippocampal LTD-to-LTP shift, which is a signature of memory impairment (Temido-Ferreira et al., 2020). Furthermore, the activation of A_2*A*_R in endothelial cells was shown to increase blood–brain barrier (BBB) permeability in mice, facilitating the penetration of macromolecules into the brain, such as proinflammatory and neurotoxic factors, which might contribute to AD pathology (Carman et al., 2011). Curiously, a gene-based association study reported that the gene encoding A_2*A*_R (ADORA2A) is associated with hippocampal volume in humans, being its minor allele, rs9608282, related with larger hippocampal volumes and better memory (Horgusluoglu-Moloch et al., 2017).

Although the above-described findings reinforce a link between A_2*A*_R and cognitive deficits associated with AD, the impact of astrocytic A_2*A*_R in AD-associated cognitive deficits has surprisingly been underexplored. In the brain of AD patients, it was reported that A_2*A*_R levels are increased in astrocytes but not in microglia (Orr et al., 2015). Moreover, cultured astrocytes exposed to Aβ_1–42_ also exhibited an upregulation of A_2*A*_R, which is related with a reduced capacity of astrocytes to uptake glutamate (Matos et al., 2012b) that can trigger excitotoxicity. This decrease in glutamate uptake is caused by the downregulation of glutamate transporter, GLT-1, in astrocytes, whose activity is dependent on Na^+^/K^+^ activity that is regulated by A_2*A*_R (Matos et al., 2013). Interestingly, the genetic lowering of GLT-1 expression in AD mice (APP/PS1) causes an earlier onset of cognitive deficits (Mookherjee et al., 2011). The upregulation of A_2*A*_R in cultured astrocytes strongly modulates the transcriptome of these cells, affecting mainly genes related with neuroinflammation, angiogenesis and cell activation; some of the changes were reversed by a selective A_2*A*_R antagonist (Paiva et al., 2019). Although there is evidence suggesting that A_2*A*_R blockade restrains astrocyte reactivity (Hindley et al., 1994; Brambilla et al., 2003; Minghetti et al., 2007, see also Cunha, 2016), the pathophysiological impact of astrocytic A_2*A*_R upsurge in reactive astrocytes in conditions of brain disorders, in particular of AD, remains to be defined. It was described that A_2*A*_R in astrocytes regulate Ca^2+^ efflux from the endoplasmic reticulum and glutamate release (Kanno and Nishizaki, 2012) and also ATP release (unpublished data from our group, Madeira D, poster #726, T05-063B, *XIV European meeting on glial cells in health and disease*) as well as GABA transport (Cristóvão-Ferreira et al., 2013), which support that astrocytic A_2*A*_R regulate the secretory capacity of these cells and, thus, impact on astrocyte-neuron communication. Therefore, taking in account the role of A_2*A*_R in controlling key astrocytic functions and the upregulation of A_2*A*_R in AD, it might be helpful to develop strategies, genetic or pharmacological, directed to tinker specifically with astrocytic A_2*A*_R to halt AD-associated cognitive decline.

## Conclusion and Future Perspectives

**FIGURE 1:**
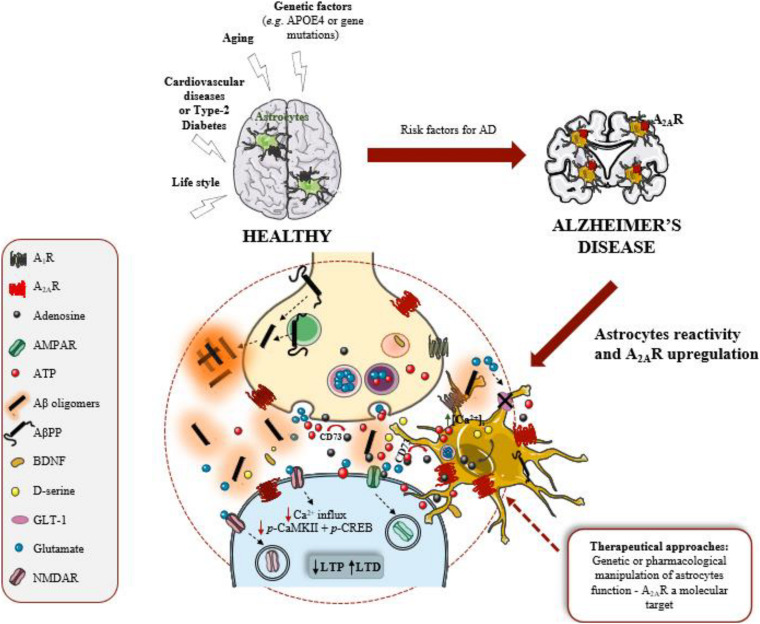
Astrocytes and A_2*A*_R are active players in Alzheimer’s disease (AD). Adenosine A_2*A*_ receptors (A_2*A*_R) are upregulated in AD brain, namely in astrocytes. These glial cells are considered the third active element of the synapse, regulating synaptic plasticity, mainly long-term potentiation (LTP) and long-term depression (LTD) that are events mediated by AMPAR and NMDAR (receptors for glutamate). Astrocytes became reactive and dysfunctional in response to amyloid-β peptide (Aβ) overproduction, derived from amyloid-β precursor protein (AβPP) proteolytic cleavage. Astrocyte dysfunction negatively impacts on synaptic plasticity, the neurophysiological basis of memory. A_2*A*_R regulate key functions of astrocytes, such as intracellular Ca^2+^ levels [Ca^2+^]_*i*_, glutamate uptake by the transporter (GLT-1) and the release of ATP, and consequently regulate synaptic plasticity. Thus, A_2*A*_R in astrocytes might be a therapeutical target to manage AD.

Although it is known for over a century that astrocytes display substantial morphological alterations in AD brains, neuronal damage has been considered the paramount pathological event causing cognitive decline. In recent years, this “neurocentic” view of AD has been changing, with the growing evidences that astrocytic morphological changes also reflect functional alterations with impact in AD pathology. Astrocytes are distributed throughout the brain in an optimal arrangement to establish chemical and physical interactions with neuronal synapses. Thus, changes in astrocyte morphology and function disturb synaptic contacts, function, and plasticity and, consequently, contribute to early cognitive deficits in AD (Figure 1). Understanding which astrocyte-neuronal signaling pathways are disrupted could lead to the development of more effective therapies as well as to the identification of novel biomarkers for synaptopathies, such as AD (Allen and Barres, 2009; Agostinho et al., 2020). Currently, a great research challenge has been the development of tools and strategies to detail the molecular pathways underlying the diverse functions of different astrocyte subpopulations, in particular of human astrocytes that are larger, more ramified and more heterogenous than rodent astrocytes. Recent studies using refined labeling strategies based on astrocytic promoters, and intersectional fluorescence-activated cell sorting-based strategy, as well as single-cell RNA sequencing provided great advances in revealing distinct spatial distributions of distinct astrocyte populations, possessing distinct morphologies and physiologies (Morel et al., 2019; Batiuk et al., 2020). However, it remains to be explored how the morphology and function of different astrocytic subpopulations are influenced by local environment, mainly by signal instigators of disease as well as the molecular signals involved in astrocyte communication with other brain cells. Filling these gaps of knowledge will set the stage for tackling astrocytic functions as targets to delay the onset of synaptic and memory deficits in AD.

Adenosine exerts two parallel modulatory roles in the brain, acting as a homeostatic modulator and also as a neuromodulator at the synaptic level. Its effects are mediated by G protein-coupled receptors, being the subtypes A_1_R and A_2*A*_R the most abundant and studied. A_2*A*_R are considered to mediate excitatory effects and to be more involved in neurodegeneration, contrasting to A_1_R (reviewed in Lopes et al., 2020). Furthermore, increasing evidences show that A_2*A*_R levels are not only upregulated in neurons but also in astrocytes, in the brain of AD patients and of AD mouse models. Moreover, it was shown that the astrocytic A_2*A*_R upregulation contributes to memory loss in AD (Orr et al., 2015). These findings are relevant in view of the convergence of epidemiological and animal studies showing that caffeine intake is inversely correlated with memory deterioration in aging and AD, an effect mimicked by the selective A_2*A*_R blockade (Cunha, 2016). As discussed in this review, we are only beginning to unveil the role of adenosine signaling in the control of astrocyte-neuron communication. However, there are already a set of evidences that reinforces the interest of exploring the therapeutic potential of astrocytic A_2*A*_R. In the future, an ambitious challenge will be getting strategies, genetic or pharmacological, directed to A_2*A*_R in astrocytes that allow control of their functions to be introduced into clinical practice as novel drugs to AD.

## Author Contributions

All the authors participated in the writing of this manuscript.

## Conflict of Interest

RC is a scientific advisor of the Institute for Scientific Information on Coffee (ISIC). The remaining authors declare that the research was conducted in the absence of any commercial or financial relationships that could be construed as a potential conflict of interest.
